# Remodelling of miRNA Regulatory Landscape During West Nile Virus (WNV) Infection

**DOI:** 10.3390/epigenomes10020041

**Published:** 2026-06-18

**Authors:** Lachlan De Hayr, Alexander A. Khromykh, Andrii Slonchak

**Affiliations:** 1 School of Chemistry and Molecular Biosciences, University of Queensland, Brisbane, QLD 4072, Australia; 2 Australian Infectious Diseases Research Centre, Global Virus Network Centre of Excellence, Brisbane, QLD 4072, Australia; 3 Systems Virology Laboratory, QIMR Berghofer Medical Research Institute, Brisbane, QLD 4072, Australia

**Keywords:** West Nile virus, orthoflavivirus, miRNA, antiviral response, miRNA regulation, Kunjin, flavivirus, systems biology, transcriptomics

## Abstract

**Background/Objectives:** West Nile virus (WNV) remains a significant threat to human health, with no approved antiviral treatments or vaccine available. A better understanding of the molecular mechanisms governing flavivirus–host interactions is needed to identify host regulatory pathways involved in infection. This study aimed to investigate how WNV infection remodels the host miRNA–mRNA regulatory landscape. **Methods:** WNV-induced changes in host miRNA expression in HEK-293 cells were profiled using miRNA-Seq. Transcriptome-wide host gene expression changes in WNV-infected cells were analysed using RNA-Seq. Gene Ontology and pathway enrichment analyses were conducted using DAVID. Integrated miRNA–mRNA network reconstruction was performed using Cytoscape based on the experimentally validated miRNA–mRNA interactions in miRNet database. **Results:** WNV infection induced global changes in host miRNA expression, with pathogenic NY99 and non-pathogenic Kunjin strains of the virus producing overlapping and strain-specific alterations in the miRNA landscape. Transcriptome analysis showed strong induction of interferon-related responses and activation of NF-κB and MAPK signalling pathways in the infected cells. In contrast, pathways associated with RNA processing, splicing, and proteasomal degradation were downregulated. Integrated miRNA–mRNA network analysis identified miR-197-3p, miR-301b-3p, miR-129-3p, miR-3662, and miR-128-5p as candidate regulatory hubs involved in WNV-induced transcriptome remodelling. These networks suggested that miRNA-mediated regulation may influence antiviral signalling, apoptosis, and RNA metabolism during infection. **Conclusions:** These findings suggest that WNV infection broadly remodels host miRNA–mRNA regulatory networks and identifies candidate miRNAs that may contribute to the regulation of antiviral and cellular stress responses. These predicted regulatory interactions provide a foundation for future experimental validation.

## 1. Introduction

West Nile virus (WNV) is a positive-sense, single-stranded RNA virus that belongs to the genus *Orthoflavivirus* within the Flaviviridae family. It is primarily transmitted to humans through the bite of infected *Culex* sp. mosquitoes, with birds serving as the main reservoir hosts. WNV infections range from asymptomatic or mild febrile illness to severe neuroinvasive disease, including meningitis and encephalitis, which can lead to long-term neurological complications or death [1,2]. Currently, there are no approved vaccines or specific antiviral therapies available to prevent or treat WNV infection [3].

In vertebrate hosts, the defence against WNV and other positive-strand RNA viruses relies primarily on protein-based interferon (IFN) response (reviewed in [4]). Host cells recognise flavivirus infections through several pathogen-sensing pathways, including retinoic-acid-inducible gene-I-like receptors (RLRs), Toll-like receptors (TLRs), and nucleotide-binding oligomerisation domain-like receptors (NLRs). These receptors trigger the signalling cascades that result in activation of the transcription factors IRF3 and IRF7, which drive the expression of interferons and proinflammatory chemokines [5,6]. Secreted interferons then bind to interferon α/β receptors (IFNARs), stimulating the transcription of interferon-stimulated genes (ISGs) that establish an antiviral state within the cell [7].

While protein-based innate immune mechanisms have been most studied to date, a growing number of studies indicate that noncoding RNAs also have an important function in regulating flavivirus–host interactions. In particular, the role of flaviviral noncoding RNAs known as subgenomic flaviviral RNAs (sfRNAs) in viral suppression of host immune responses has been well-established [8,9,10,11,12,13]. In addition, emerging evidence suggests that host miRNAs are also involved in modulating antiviral responses and virus–host dynamics in flavivirus-infected cells [14,15].

miRNAs represent the most extensively studied classes of small noncoding RNAs due to their wide range of functions in development and cancer, as well as their potential as biomarkers and therapeutic targets [16,17,18,19]. These short RNA molecules, typically 19–21 nucleotides in length, are generated by endonucleolytic processing of longer RNA transcripts and then incorporated into the RNA-induced silencing complex (RISC), where they associate with Argonaute (Ago) proteins [20,21,22,23]. RISC is guided to complementary sequences within the target mRNAs, which leads to cleavage of mRNAs by Ago or inhibition of their translation [24,25,26,27]. Translationally inactive mRNA ultimately undergo degradation following deadenylation and decapping [28,29].

Several studies have demonstrated that miRNAs contribute to flavivirus–host interactions. For instance, human miR-532-5p is upregulated upon infection with Kunjin strain of WNV and inhibits expression of the host gene SESTD1, which is required for viral replication [14]. In vector mosquito species *Aedes aegypti*, miR-2940-5p modulates viral replication by altering the expression of the metalloprotease gene [15]. In dengue virus (DENV) infection, miR-155 was shown to be upregulated to enhance antiviral immune responses by promoting type I interferon signalling and inflammatory cytokine production [30]. The miRNA miR-146a, a negative regulator of TRAF6 and IRAK1 in the NF-κB signalling pathway, has been shown to facilitate DENV replication by suppressing antiviral responses [31] and inhibiting autophagy in infected cells [32]. In addition, miR19a-3p has been identified as virus-permissive host factor for DENV Zika virus (ZIKV) infection [33]. Furthermore, in ZIKV-infected cells, miR-21, miR-34a, and miR-155 are induced in neural progenitor cells and suppress apoptosis and innate immune signalling, promoting survival of ZIKV-infected cells and impacting neurodevelopmental outcomes [34,35]. In Japanese encephalitis virus (JEV) infection, miR-146a [36] and miR-29b [37] have been shown to modulate microglial activation and interferon responses. Collectively, these studies demonstrate that specific host miRNAs can act either as antiviral effectors or as proviral modulators by targeting key immune regulators. This highlights the importance of understanding miRNA dynamics during viral infection as a potential foundation for identifying novel targets for the antiviral strategies.

In this study, we employed small RNA sequencing to characterize changes in miRNA expression following WNV infection of human HEK-293 cells. We compared infections with the highly pathogenic NY99 strain (WNV_NY99_) and the non-pathogenic for humans Kunjin strain (WNV_KUN_) to identify both common and strain-specific miRNA responses. Integration of miRNA and mRNA expression data revealed potential miRNA-regulated gene networks affected by infection. Functional-enrichment analysis indicated that differentially expressed miRNAs potentially influence host pathways involved in stress response, apoptosis, RNA processing, and splicing. Together, our findings demonstrate that WNV-induced alterations in the miRNA landscape can broadly impact mRNA processing and cellular stress responses during infection.

## 2. Results

### 2.1. WNV Infection Substantially Alters the Host miRNA Expression Landscape

To examine the effects of WNV infection on host miRNA expression, HEK-293 cells were infected with either WNV_NY99_ or the WNV_KUN_ at MOI = 1 and analysed by small RNA sequencing at 48 h post-infection (hpi). WNV exhibits broad tissue tropism and is capable of replicating in a wide range of cell types, including epithelial, endothelial, and fibroblast, particularly during the systemic phase of infection. Notably, WNV can infect kidneys, causing acute renal failure and potential long-term chronic kidney disease (CKD) [38,39,40]. Therefore, the HEK-293 human embryonic kidney epithelial cell line was selected, as it is representative of kidney cellular environment and is known to support WNV replication [41,42].

**Figure 1:**
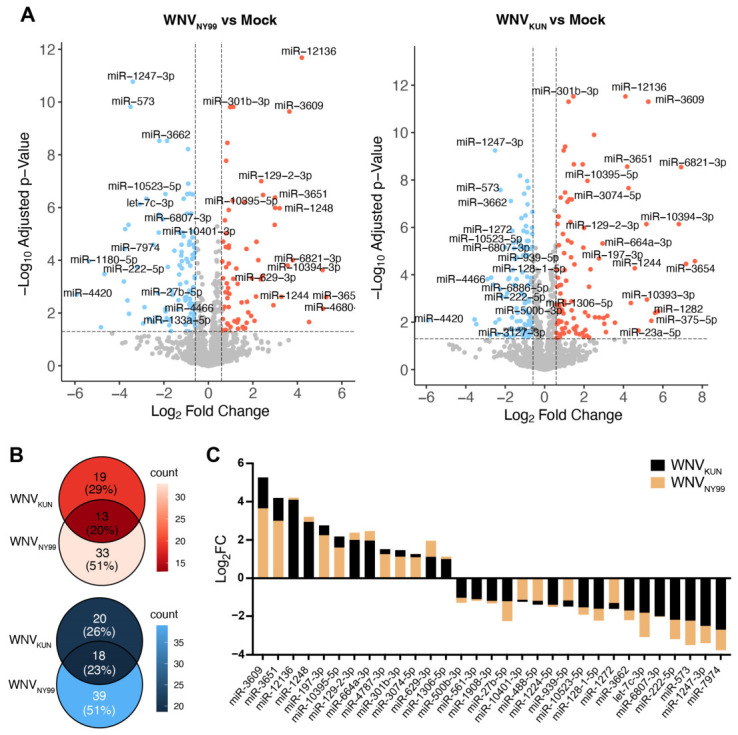
NY99 and Kunjin strains of WNV induce profound and overlapping changes in miRNA profiles of HEK-293 cells. (**A**) Differentially expressed miRNAs in HEK-293 cells infected with WNV_NY99_ or WNV_KUN_ at MOI = 1. Points in blue represent significantly downregulated miRNAs (LogFC < −1, FDR-adjusted *p*-value < 0.05), and points in red represent significantly upregulated miRNAs (LogFC > 1; FDR-adjusted *p*-value < 0.05). (**B**) Venn diagrams showing the overlapping profiles of significantly downregulated (blue) and upregulated (red) miRNAs in WNV_NY99_- and WNV_KUN_-infected HEK-293 cells. (**C**) Expression changes of individual miRNAs commonly regulated during WNV_NY99_ and WNV_KUN_ infection. Bars represent mean Log_2_ fold-change values (relative to mock-infected cells) calculated from three biological replicates of HEK-293 cells infected with WNV_NY99_ (gold) or WNV_KUN_ (black).

We found that both WNV strains induced substantial changes in the cellular miRNA landscape (Figure 1A, Supplementary Tables S1 and S2). Although both viruses replicated in HEK-293 cell at the comparable levels (Supplementary Figure S1), the Kunjin strain elicited a more diverse miRNA response (Figure 1B,C). WNV_NY99_ infection led to the differential expression of 48 miRNAs (19 upregulated, 29 downregulated), whereas WNV_KUN_ infection altered 70 miRNAs (32 upregulated, 38 downregulated) (Figure 1B). Comparative analysis revealed 13 commonly upregulated and 18 commonly downregulated miRNAs between the two WNV strains (Figure 1B), with fold-change magnitudes remaining largely consistent across strains (Figure 1C).

Among the most upregulated miRNAs in WNV_NY99_-infected cells were miR-12136, miR-3609, miR-1248, miR-301b-3p, miR-129-3p, and miR-197-3p (Figure 1A,C). In WNV_KUN_ infection, a similar pattern was observed, with miR-12136, miR-3609, miR-1248, miR-301b-3p, miR-129-3p, and miR-197-3p among the top upregulated miRNAs (Figure 1A,C). Conversely, miR-1247, miR-573, miR-3662, and miR-222-5p were among the most downregulated miRNAs in both infections, displaying log2 fold changes below −2 (Figure 1A,C). Several of these miRNAs have documented roles in regulating innate immune signalling and virus–host interactions. miR-129-2-3p has been reported to modulate inflammatory and antiviral responses by targeting components of NF-κB and STAT signalling pathways and influencing cytokine production during viral infection [43]. miR-301b-3p is a known regulator of innate immune activation, acting through suppression of negative regulators of NF-κB signalling such as PTEN and NKRF, which can amplify pro-inflammatory and antiviral gene expression and inhibit apoptosis [44,45]. miR-197-3p has been implicated in immune regulation and viral pathogenesis, in part through targeting genes involved in interferon and inflammatory signalling and has been shown to enhance HIV-1 replication by targeting DDX52 and stabilising Vif protein expression [46]. In addition, miR-1248 has been demonstrated to promote type I interferon induction by directly activating RIG-I signalling in noncanonical manner, leading to enhanced IFN-β production. The roles of other highly regulated miRNAs, such as miR-12136 and miR-3609, remain poorly understood.

The absolute expression levels of the identified commonly differentially expressed miRNAs ranged from approximately 4 counts per million (CPM) to more than 1500 CPM in the highest expressing experimental group (Supplementary Figure S2). Among these, let-7c-3p was the least abundant, with the expression level of less than 4 CPM (Supplementary Figure S2). In contrast, miR-301b-3p exhibited the highest overall abundance, rising from 681 CPM in mock to 1829 CPM in WNV_KUN_-infected cells (Supplementary Figure S2). Most of other identified miRNAs were expressed within the 10–100 CPM range (Supplementary Figure S2), a level at which numerous miRNAs have previously been reported to exert biologically meaningful regulatory effects [47,48,49,50]. While highly abundant miRNAs are generally expected to have strong regulatory impact due to greater target occupancy, numerous studies indicate that moderately or even lowly expressed miRNAs often are also biologically significant [47,50,51]. Functional impact depends not only on absolute abundance but also on factors such as miRNA-target stoichiometry, binding affinity, and target transcript abundance [52]. In particular, miRNAs regulating low-abundance mRNAs [53] or transcripts encoding key regulatory proteins, such as transcription factors, can produce substantial biological effects despite relatively modest expression levels [54].

In summary, the results of the miRNA sequencing analysis demonstrate that WNV infection substantially reprograms host miRNA expression, with both strains inducing overlapping as well as strain-specific changes in miRNA levels. The consistent regulation of a subset of miRNAs across both viral strains suggests a conserved host response mechanism that may affect antiviral signalling and stress adaptation pathways.

### 2.2. WNV Infection Induces Activation of Antiviral and Stress Responses and Suppression of the Pathways Associated with Catabolism and RNA Homeostasis

**Figure 2:**
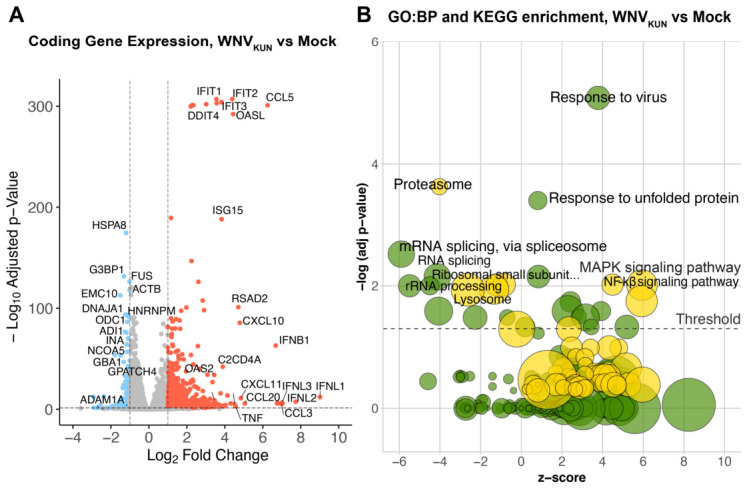
WNV_KUN_ infection of HEK-293 cells induces antiviral and stress-response genes while suppressing RNA and protein metabolism pathways. (**A**) Differentially expressed genes (DEGs) in WNV_KUN_-infected HEK-293 cells. Cells were infected in triplicates with WNV_KUN_ at MOI = 1 or mock-infected. Total RNA was isolated at 48 hpi and analysed by RNA-Seq following poly(A)-enrichment. Points in blue represent significantly downregulated genes (LogFC < −1, FDR-adjusted *p*-value < 0.05), and points in red represent significantly upregulated genes (LogFC > 1, FDR-adjusted *p*-value < 0.05). (**B**) Gene Ontology Biological Processes (green) and KEGG pathway (yellow) enrichment analyses of DEGs identified in WNV_KUN_-infected HEK-293 cells. Positive z-scores indicate activation of biological processes or KEGG pathways, while negative z-scores indicate inhibition. Circle size corresponds to the number of genes associated with each GO or KEGG term.

To investigate how the miRNAs identified in our study contribute to the regulation of host gene expression during WNV infection, we performed RNA-Seq analysis of the coding transcriptome in infected and uninfected HEK-293 cells. Transcriptome-wide mRNA profiling was performed for the Kunjin strain, which served as the representative WNV strain. The differential gene expression analysis revealed profound changes in cell transcriptome following WNV_KUN_ infection, with a predominant trend towards upregulation rather than repression of the host gene expression (Figure 2A, Supplementary Table S3). Notably, several canonical antiviral response genes, including *IFNB1* (interferon-β), *IFNL1* (interferon-λ), and members of the interferon-induced proteins with tetratricopeptide repeats (*IFIT*) family, were strongly induced (Figure 2A), indicating for robust type I and type III interferon-mediated immune responses.

The Gene Ontology (GO)-enrichment analysis of the differentially expressed genes (Figure 2B) revealed activation of biological processes associated with response to virus and the unfolded protein response (UPR), which is consistent with activation of innate immune and cellular stress pathways. Conversely, downregulated biological processes were related to RNA splicing, mRNA processing, and ribosomal RNA metabolism (Figure 2B), suggesting a virus-induced suppression of host transcriptional and translational machinery, which may reflect a shift in resource utilisation from host to virus or a viral evasion strategy targeting host RNA maturation.

KEGG pathway analysis (Figure 2B) further supported these findings, revealing activation of the NF-κB and MAPK signalling pathways, which are known to be involved in initiating inflammatory and immune responses [55,56]. In contrast, pathways associated with proteasome and lysosome functions were suppressed, indicating that WNV infection inhibits cellular catabolic pathways, likely to prevent degradation of viral proteins.

Collectively, the results of the transcriptomic profiling reveal that WNV infection triggers a range of host responses characterised by strong activation of antiviral and stress-related pathways coupled with suppression of cellular RNA processing and catabolic functions, which is consistent with the transcriptional responses previously described in human cells infected with other flaviviruses [8,10].

### 2.3. Network Reconstruction Analysis Identified miRNAs 197-3p, 301b-3p, 129-2-3p, 3662 and 128-1-5p as Candidate Regulators in miRNA-Mediated Response to WNV Infection

To elucidate how miRNAs may contribute to the host responses observed during WNV infection, we integrated small RNA-Seq and mRNA-Seq data to reconstruct miRNA–mRNA interaction networks. In this analysis, we focused on miRNAs commonly differentially expressed in response to both viral strains to identify the conserved signatures of miRNA regulation in WNV infection and used WNV_KUN_-derived coding transcriptome data as a reference. Differentially expressed miRNAs commonly identified in WNV_KUN_- and WNV_NY99_-infected HEK-293 cells were analysed using the miRNet 2.0 software to reveal their experimentally validated mRNA targets. Following the canonical model of miRNA-mediated regulation, where increased miRNA expression suppresses target mRNA levels and decreased miRNA expression relieves this repression, we filtered identified miRNA–mRNA pairs according to inverse expression relationships. Specifically, only mRNAs showing reduced expression were retained as putative targets of upregulated miRNAs and vice versa. To reduce the number of interactions for analytical tractability, we further filtered the miRNA–mRNA interaction matrix to included only mRNAs targeted by two or more differentially expressed miRNAs, as effective gene repression typically requires cooperative action of multiple miRNAs [57,58].

**Figure 3:**
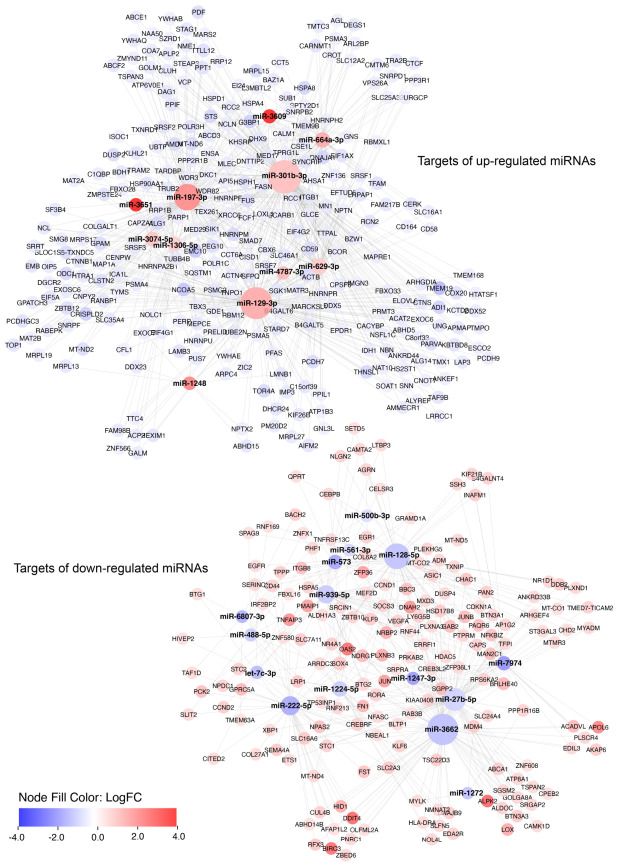
Predicted miRNA–mRNA interaction networks in WNV_KUN_-infected HEK-293 cells. Up-regulated (top panel) and down-regulated (bottom panel) miRNAs commonly affected by WNV_KUN_ and WNV_NY99_ (Figure 1C) were integrated with RNA-Seq-derived mRNA expression data to reconstruct miRNA–mRNA interaction networks using the miRNet 2.0 platform which employs the database of experimentally validated miRNA targets. Only interactions showing inverse expression relationships and involving mRNAs targeted by two or more miRNAs were retained for network construction. Networks were visualised in Cytoscape. Node colour represents log2 fold change and node size corresponds to betweenness centrality, indicating the relative regulatory influence of each node.

These filtered interactions were assembled into the miRNA–mRNA network using Cytoscape. The computational analysis was then performed to quantitatively characterise the weight of each miRNA in the network. The betweenness centrality parameter was calculated for each miRNA node, quantifying the number of shortest paths in the network that pass through that node and thus reflecting its potential role as a regulatory hub or bottleneck in the interaction network. The resulting networks were plotted as directed graph diagrams, with node sizes reflecting betweenness centrality values and node colour representing mRNA expression fold change with upregulated miRNAs having 294 nodes and 723 edges and downregulated miRNAs having 185 nodes with 407 edges (Figure 3, Supplementary Table S4).

In the resulted network, miR-197-3p, miR-301b-3p, and miR-129-3p exhibited the highest betweenness centrality, which indicates for their central role within the miRNA–mRNA interaction network (Figure 3). For downregulated miRNAs, we identified fewer interacting mRNAs with miR-3662 and miR-128-5p linked to the most targets (Figure 3). Notably, these two miRNAs were among the most downregulated miRNAs suggesting they may release negative control of multiple mRNAs following infection.

**Figure 4:**
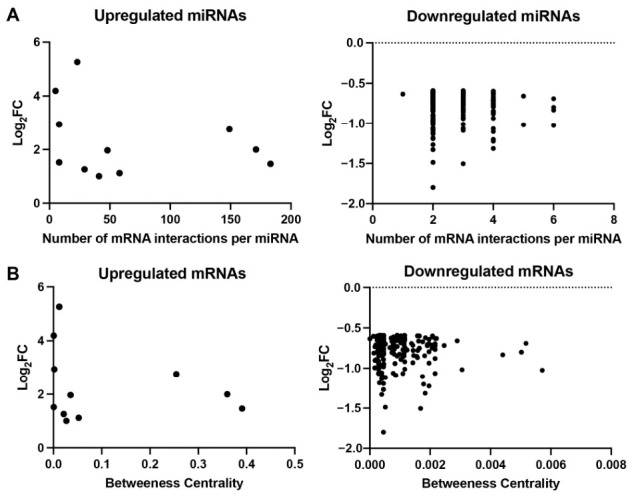
Network connectivity of differentially expressed miRNAs and mRNAs in WNV-infected cells is independent of their expression magnitude. (**A**) Scatter plot showing the relationship between miRNA fold change and the number of differentially expressed mRNA targets for each miRNA. (**B**) Scatter plot showing the relationship between mRNA expression levels and the number of miRNAs predicted to target each transcript, indicating no significant association between transcript abundance and miRNA targeting density. Correlation was determined using Spearman’s correlation (R) with associated *p*-value (*p*).

We next examined whether the number of mRNA interactions for each miRNA influenced the degree of regulatory control exerted by miRNAs on their targets. To test this, we plotted miRNA fold change against the number of mRNA targets and performed correlation analysis (Figure 4A). The analysis revealed no apparent correlation (Upregulated miRNAs; R = −0.3827, *p* = 0.1219 and Downregulated miRNAs; R = −0.3182, *p* = 0.1708), indicating that miRNAs with higher fold-change did not necessarily regulate a greater or smaller number of target transcripts compared to those with lower fold changes. Similarly, correlation analysis between mRNA expression levels and the number of different miRNAs predicted to bind each transcript (Figure 4B) showed no relationship, suggesting that the magnitude of mRNA expression change was not determined by the number of miRNAs targeting it.

In summary, the network reconstruction analysis predicted a broad network of miRNA-regulated genes during WNV infection. It also suggests that several miRNAs such as miR-197-3p, miR-301b-3p, miR-129-3p, miR-3662, and miR-128-5p likely act as central regulatory hubs in this network. In addition, the lack of correlation between expression changes and interaction counts suggest the importance of network topology over expression magnitude in defining the regulatory influence of these miRNAs in response to WNV.

### 2.4. miRNA-Mediated Regulation Is Predicted to Modulate Host Gene Transcription and Stress Responses During WNV Infection

**Figure 5:**
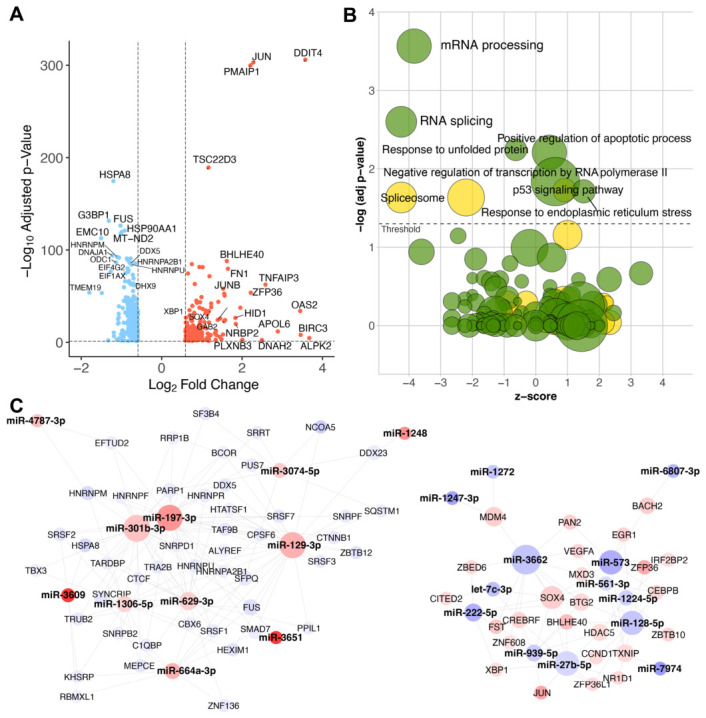
Differentially expressed target genes of miRNAs affected by WNV infection are involved in apoptosis, stress response and RNA metabolism. (**A**) Differential expression of host genes identified as targets of infection-affected miRNAs in WNV_KUN_-infected HEK-293 cells compared to mock. Points in blue represent significantly downregulated genes (LogFC < −1, FDR-adjusted *p*-value < 0.05), and points in red represent significantly upregulated genes (LogFC > 1, FDR-adjusted *p*-value < 0.05). (**B**) Gene Ontology Biological Processes and KEGG pathway enrichment analyses of DEGs shown in (**A**). Positive z-scores indicate activation negative z-scores indicate inhibition. GO terms are shown in green and KEGG pathways are shown in yellow. Circle size corresponds to the number of genes associated with each GO or KEGG term. (**C**) miRNA–mRNA interaction networks reconstructed for the genes associated with GO term “RNA processing” in (**B**). Network for upregulated miRNAs is shown on the left and for downregulated miRNAs is on the right. Nodes are coloured based on their Log2 fold change, and node sizes are scaled based on their betweenness centrality values.

To obtain further understanding of the potential biological impact of miRNA modulation during WNV infection, we performed Gene Ontology-enrichment analysis specifically on the differentially expressed genes identified as miRNA targets in the reconstructed interaction networks. From the list of identified miRNA targets, we selected mRNAs showing significant expression changes (in either direction) and subjected them to GO and KEGG pathway analyses (Figure 5A,B).

Notably, downregulated targets exhibited relatively modest decreases in expression, whereas upregulated targets displayed greater variability and larger fold changes (Figure 5A). Activated biological processes associated with miRNA targets included regulation of apoptosis, response to endoplasmic reticulum stress, negative regulation of RNA polymerase II transcription, and activation of the p53 signalling pathway (Figure 5B). This suggests that in uninfected cells, miRNAs normally act to suppress apoptotic and stress-related pathways, whereas during infection, the downregulation of these miRNAs may relieve this repression, promoting transcriptional arrest and apoptosis of infected cells as a part of the host antiviral response. However, the relatively sparse number of interactions linked to these upregulated processes (Figure 5C) indicates that the overall miRNA-mediated influence on these pathways may be limited. In contrast, the downregulated pathways, including mRNA processing, RNA splicing, and the spliceosome pathway, are characterised by dense interconnectivity between miRNAs and their targets (Figure 5C). This suggests that these processes are likely under strong miRNA-mediated control, potentially reflecting a coordinated suppression of the host’s transcriptional and RNA maturation machinery during WNV infection.

Collectively, the results of transcriptomic profiling and miRNA–mRNA network reconstruction indicate that infection-induced alterations in miRNA expression may be associated with suppression of host transcriptional and RNA-processing capacity, as well as enhancement of cellular stress and apoptotic responses. This suggests that perturbation to the host miRNA landscape during WNV infection likely occur as a part of host antiviral defence activation as well as virus-driven remodelling of the intracellular environment to support replication.

## 3. Discussion

MicroRNAs have crucial functions in regulating host processes through the modulation of gene expression and are known to be differentially expressed in many pathological conditions [59,60]. The roles of miRNAs in regulating antiviral responses and the capacity of viruses to manipulate host miRNA expression to modulate immune signalling and alter cellular homeostasis are also becoming increasingly recognised [61,62,63]. Although a number of studies previously examined the role of individual miRNAs in viral infections, this reductionist approach does not capture the complex, systems-level miRNA regulatory networks that may operate during infection. In this study, we utilised small RNA sequencing to identify miRNA expression changes in response to WNV infection and integrated the miRNA expression data with transcriptome-wide mRNA profiling to elucidate the potential system-level effects of miRNAs on host gene regulation during infection. This integrative approach revealed that WNV infection results in widespread changes in both miRNA and mRNA expression and provided evidence that miRNA-mediated regulation may influence host pathways involved in RNA processing, transcription, stress response, and apoptosis.

The gene expression profiling of the infected cells revealed that WNV infection triggers a strong interferon-mediated antiviral response, characterised by the induction of *IFNB1*, *IFNL1*, and multiple interferon-stimulated genes (ISGs). This is consistent with current knowledge regarding the important role of type I and III IFN responses in host defence against WNV [64,65]. In line with earlier reports [66,67], we also observed activation of the MAPK and NF-κB pathways, which are central mediators of innate immune and inflammatory responses. At the same time, the infection was associated with suppression of pathways linked to ribosomal RNA processing and proteasomal degradation. The observed downregulation of ribosome and proteasome pathways has been reported in other flavivirus infections, although whether this represents a host-driven restriction to limit viral protein synthesis or a virus-induced shutdown of host translation remains a subject to discussion [68,69,70]. Overall, these results demonstrate a coordinated transcriptional reprogramming of host cells during WNV infection with activation of antiviral signalling and suppression of RNA and protein metabolism.

By integrating miRNA and mRNA expression data, we identified several miRNAs with high regulatory potential during WNV infection, including miR-197-3p, miR-301b-3p, miR-129-2-3p, miR-3662, and miR-128-5p. Network analysis revealed that miR-197-3p, miR-301b-3p, and miR-129-3p exhibited the highest betweenness centrality values, suggesting that they may act as central nodes within the WNV-induced miRNA–mRNA interaction network. Previous studies have shown that miR-197-3p suppresses type I interferon signalling and modulates inflammatory responses through regulation of TNF signalling [71,72]. In addition, it has also been implicated in enhancing HIV-1 replication by supressing the expression of antiviral gene *DDX52* [73]. miR-301b-3p has been shown to inhibit inflammatory activation by targeting *c-Myb* and by downregulating *TLR4* and *PIK3CB*, thereby reducing production of cytokines such as IL-4 and TGF-β1 [44,74,75]. Notably, miR-128-5p was among the most strongly downregulated miRNAs following infection, consistent with findings from Zika virus studies in which this miRNA was shown to activate TLR8 and the NF-κB pathway [76]. Collectively, our results and current knowledge about the functions of these miRNAs suggest that their combined action may be associated with suppression of antiviral inflammation and interferon signalling, potentially influencing the environment for WNV replication. This model is consistent with previous reports of viral inhibition of inflammation that creates more favourable conditions for virus replication [77,78].

To extend our understanding of miRNA-regulated processes during WNV infection beyond the functions of the individual miRNAs, we perform integration of miRNA and mRNA expression and reconstruction of the miRNA-regulated networks. Analysis of the validated miRNA targets among the DEGs in WNV-infected cells revealed that genes involved in mRNA processing, RNA splicing, and spliceosome assembly were significantly downregulated, while being the targets of the up-regulated miRNAs. These processes are critical for maintaining proper gene expression and host defence, and their inhibition is known to benefit RNA viruses by reducing the efficiency of antiviral gene transcription and mRNA maturation [24,79]. Notably, one of the identified down-regulated miRNA targets was heterogeneous nuclear ribonucleoprotein M (hnRNPM). Heterogeneous nuclear ribonucleoproteins (hnRNPs), including hnRNPM, have been reported to possess antiviral activity against RNA viruses and their depletion has been shown to enhance flavivirus replication [80,81,82]. Together with the results of GO-enrichment analysis of the identified miRNA targets this suggests that WNV-induced miRNA changes may be associated with suppression of host RNA processing, potentially limiting the production of antiviral transcripts and altering the cellular environment during viral replication.

Despite this apparent virus-favouring shift, certain miRNA-associated changes may reflect host-driven responses aimed at restricting infection. Gene ontology and pathway enrichment analyses of the identified miRNA targets revealed their association with upregulated p53 signalling, ER stress response, and apoptotic pathways, consistent with a programmed elimination of the infected cells through apoptotic cell death. Additionally, we identified a potential miRNA-mediated downregulation of *KHSRP*, a known negative regulator of interferon signalling, which could enhance the antiviral state of infected cells [83].

Together, our data provide a model in which WNV infection is associated with changes in miRNA–mRNA interactions that may influence host regulatory programs. On one hand, the virus appears to exploit miRNA pathways to suppress host transcriptional and RNA processing functions, thereby restricting antiviral gene expression. On the other, host cells respond by activating stress-related and apoptotic pathways as part of an intrinsic defence mechanism. The balance between these opposing pressures likely determines whether the infection proceeds toward viral persistence or clearance. In this model, miRNAs are acting as regulatory intermediates that can be co-opted by both the virus and the host.

Although, our study focused primarily on the miRNAs affected by both viral strains, the analysis of differentially expressed miRNAs revealed that Kunjin infection was associated with a greater number of altered miRNAs compared with NY99 infection. Although these strains are closely related genetically, they elicit distinct pathogenic outcomes, which may be associated with differences in host miRNA responses. Notably, WNV_KUN_ infection uniquely induced upregulation of miR-124-3p (Supplementary Figure S3), which was previously shown to be increased during Japanese encephalitis virus (JEV) infection and associated with attenuated viral replication [84]. Kunjin strain infection also exclusively upregulated miR-215-5p (Supplementary Figure S3), which has been reported to negatively regulate inflammasome activation [85,86] and enhance the hepatitis C virus (HCV) replication by targeting TRIM22 and supressing NF-κB signalling [87]. In contrast, WNV_NY99_ infection uniquely upregulated miR-3150b-3p, a potent inhibitor of the transcription factor Mash1 expression. Reduced Mash1 activity has been shown to impair differentiation of neuronal progenitor cells into mature neuronal subtypes, leading to loss of specific cell populations [88]. Additionally, WNV_NY99_ infection resulted in exclusive downregulation of miR-151a-5p and miR-181a/b-3p (Supplementary Figure S3). miR-151a-5p has been implicated in suppression of ATP production [89,90], potentially increasing cellular resources available for viral replication, whereas miR-181a/b-3p play critical roles in CD4^+^ T-cell responses, with reduced expression associated with impaired viral clearance in patients [91,92]. Collectively, these strain-specific miRNA signatures and their known functional associations may suggest the role of miRNA regulation in shaping the virulence of different viral strains.

Overall, our integrative analysis demonstrates that WNV infection induces widespread perturbations in the host miRNA–mRNA regulatory network. It also identifies candidate miRNAs involved in virus-induced perturbations of the host transcriptional landscape. Despite providing new systems-level insights into the miRNA-mediated regulation of host responses to WNV infection, this study has several limitations. First, the experiments were performed in a single human cell line (HEK-293), which was derived from embryonic kidney cells of epithelial origin. Considering that WNV has broad tissue tropism, the use of the single cell type may not fully recapitulate the full spectrum of tissue-specific WNV-induced miRNA perturbations during infection in vivo. Additionally, while our analyses integrated miRNA and mRNA expression profiles and used the database of the experimentally validated miRNA–mRNA interactions, they relied on computational modelling and correlation rather than direct experimental validation of miRNA–mRNA interactions during WNV infection. Functional assays, such as miRNA mimic and inhibitor experiments and/or Ago-RNA immunoprecipitation, will be necessary to confirm the regulatory roles of the identified miRNAs. Finally, because the present dataset profiles wild-type WNV infection, it captures the integrated host response rather than the contribution of any single upstream viral process. Dissecting the extent to which individual viral proteins, subgenomic flaviviral RNA (sfRNA) [93] and other factors drive the observed miRNA–mRNA remodelling will require isogenic viral mutants and represents an important direction for future work. Addressing these limitations in future studies will further clarify the mechanisms by which miRNA regulation shapes virus–host interactions during WNV infection.

In summary, our integrative analysis demonstrates that WNV infection induces widespread perturbations in the host miRNA–mRNA regulatory network. It also identifies candidate miRNAs involved in virus-induced perturbations of the host transcriptional landscape. Further studies aimed at experimentally validating these interactions and elucidating their mechanistic roles in infection will be required to fully close the gap in our understanding of the roles of miRNAs in flavivirus–host interactions.

## 4. Materials and Methods

**Cell culture and infection.** HEK-293 cells (CRL-1573) were obtained from ATCC and maintained in Dulbecco’s Modified Eagle Medium (DMEM; Gibco, Waltham, MA, USA) supplemented with 10% foetal bovine serum (FBS; Bovogen, Melbourne, Australia) and 1% sodium pyruvate (Gibco, Waltham, MA, USA). Prior to infection, cells were seeded into 24-well plates at a density of 1 × 10^5^ cells per well. The following day, cells were infected with Kunjin (WNV_KUN_) or New York 99 (WNV_NY99_) strain of West Nile virus (WNV) at a multiplicity of infection (MOI) of 1 (MOI = 1). After 1 h of incubation, the inoculum was removed, and the cells were washed three times with 1 mL of phosphate-buffered saline (PBS) and then cultured for 48 h in DMEM supplemented with 2% FBS.

**Viruses used in the study.** The West Nile virus Kunjin strain (New South Wales 2011) was obtained as previously described by Frost et al., and NY99 strain was obtained as isolates as described previously [94,95]. Viral stocks were propagated by infecting Vero 76 cells at an MOI = 0.1 and incubating for 3 days. Culture supernatants were harvested, clarified by centrifugation at 500× *g* for 5 min, aliquoted, and stored at −80 °C. Viral titres were determined by immunoplaque assay (IPA) as previously reported [96].

**RNA Isolation.** Total RNA was extracted from cells using TRI Reagent (Sigma-Aldrich, St. Louis, MO, USA) according to the manufacturer’s protocol. Briefly, cells were lysed directly in TRI Reagent, mixed with chloroform, and centrifuged to separate phases. The aqueous phase was collected, and RNA was precipitated with isopropanol, washed with 70% ethanol, and air-dried before resuspension in nuclease-free water. RNA concentrations were measured using a Qubit fluorometer (Thermo Fisher Scientific, Waltham, MA, USA).

**mRNA sequencing.** RNA samples for bulk RNA sequencing were extracted using TRI Reagent as described above. RNA integrity was assessed using TapeStation 4200 (Agilent, Santa Clara, CA, USA). Sequencing libraries were prepared using the Illumina TruSeq RNA Library Preparation Kit (Illumina, San Diego, CA, USA) following the manufacturer’s instructions. Library fragment sizes and concentrations were verified using the 4200 TapeStation System, and libraries were pooled and sequenced on an Illumina NovaSeq 2000 platform at the Australian Genome Research Facility (AGRF, Melbourne, Australia). Raw sequencing reads were trimmed to remove adapter sequences and reads shorter than 15 nucleotides using Cutadapt (v4.1) [97]. Processed reads were aligned to the human reference genome (GRCh38) using STAR aligner (v2.7.11b), and transcript abundance was estimated with RSEM (v1.3.0). Transcript-level counts were aggregated to gene-level expression using the tximport R package (v1.37.2). Differential gene expression analysis was performed using DESeq2 (v3.21) [98,99].

**Small RNA sequencing.** Small RNA fractions were isolated from HEK-293 cells using the miRVana miRNA Isolation Kit (Ambion, Waltham, MA, USA) following the manufacturer’s protocol. Libraries for small RNA sequencing were prepared using the Illumina TruSeq Small RNA Library Kit and sequenced on the Illumina MiSeq platform at AGRF (Melbourne, Australia). Adapter trimming was performed using Cutadapt (v4.1), and reads were aligned to the human genome (GRCh38) using Bowtie (v1.2.0) [97,100]. miRNA quantification was performed using FeatureCounts (v2.1.1) with the miRBase human miRNA GFF3 annotation. Differential expression analysis of miRNAs was conducted using edgeR (v3.36.0) [101,102].

**miRNA-target network reconstruction.** miRNAs displaying a log_2_ fold change above the defined threshold (log_2_FC ≥ 1 or ≤−1) in both Kunjin- and NY99-infected cells were used for target prediction analysis with the online tool miRNet 2.0 [103]. miRNet integrates validated miRNA–mRNA interaction data derived from experimentally supported databases. Identified target mRNAs were filtered to include only those exhibiting a fold change greater than 1.5 or less than −1.5, in RNA-Seq datasets. Further refinement retained mRNAs containing binding sites for two or more of the identified miRNAs. Two interaction networks, representing upregulated and downregulated miRNAs and their predicted targets, were visualised using Cytoscape (v3.10.3) software [104]. Nodes were coloured according to fold-change values, and node size was scaled by betweenness centrality calculated in Cytoscape.

**Gene Ontology (GO) and KEGG pathway analysis.** Genes showing a fold change ≥ 1.5 from bulk RNA-Seq differential expression analyses were subjected to functional-enrichment analysis using DAVID [105]. GO term and KEGG pathway analyses were performed using thresholds of count = 5, EASE = 0.1, and *p*-value correction by the Benjamini–Hochberg method. Similarly, genes identified as miRNA–mRNA interaction targets were analysed for enriched GO terms and pathways using thresholds of count = 2 and EASE = 0.1. DAVID outputs were integrated with differential expression data to calculate Z-scores using the R package GOPlot (v1.0.2) [106].

**Data visualisation.** Data visualisation was conducted in R using the packages EnhancedVolcano (v1.11.3), ggplot2 (v3.5.2), and GOPlot (v1.0.2). Volcano plots of differential gene expression were generated using EnhancedVolcano. Overlapping miRNAs between datasets were illustrated with GoVenn, bar charts were produced with ggplot2, and bubble plots for enriched GO terms were generated using custom scripts based on GOPlot [106,107].

## Data Availability

All data supporting this study are available within the article and Supplementary Materials or upon request from the authors. Raw RNA-Seq data have been deposited in BioProject database with IDs PRJNA1425236 for the small RNA and PRJNA142903 for mRNA data.

## Supplementary Materials

The following supporting information can be downloaded at: https://www.mdpi.com/article/10.3390/epigenomes10020041/s1, Table S1: Differentially expressed miRNAs in HEK-293 cells infected with Kunjin strain of WNV compared to mock; Table S2: Differentially expressed miRNAs in HEK-293 cells infected with NY99 strain of WNV compared to mock; Table S3: Differentially expressed genes in HEK-293 cells infected with Kunjin strain of WNV compared to mock; Table S4: Number of connected edges and the betweenness centrality of miRNAs in the reconstructed network maps; Figure S1: Replication levels of WNV_NY99_ and WNV_KUN_ in HEK-293 cells as evident from viral RNA short read counts; Figure S2: Expression levels of miRNA commonly differentially expressed in HEK-293 cells infected with WNV_KUN_ and WNV_NY99_; Figure S3: Expression levels of miRNA uniquely differentially expressed in HEK-293 cells infected with either WNV_KUN_ or WNV_NY99_ compared to mock-infected cells.
