# Supplementary material for: Remodelling of miRNA Regulatory Landscape During West Nile Virus (WNV) Infection
Source: Epigenomes. 2026 Jun 18;10(2):41. doi: 10.3390/epigenomes10020041 (PMC13298079; doi:10.3390/epigenomes10020041)
Supplement: Supplementary file 1 [file epigenomes-10-00041-s001.zip › Supplementary Figures.pdf]

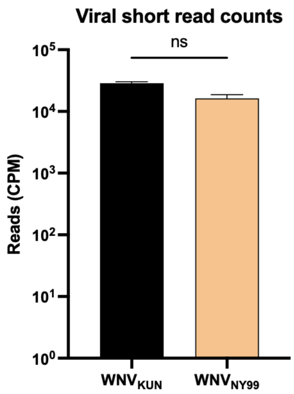

Supplementary figure S1. Replication levels of WNV_{NY99} and WNV_{KUN} in HEK-293 cells as evident from viral RNA short read counts. HEK-293 cells were infected with each virus at MOI=1. At 48hpi total RNA was isolated from the infected cells and analysed by RNA-Seq.

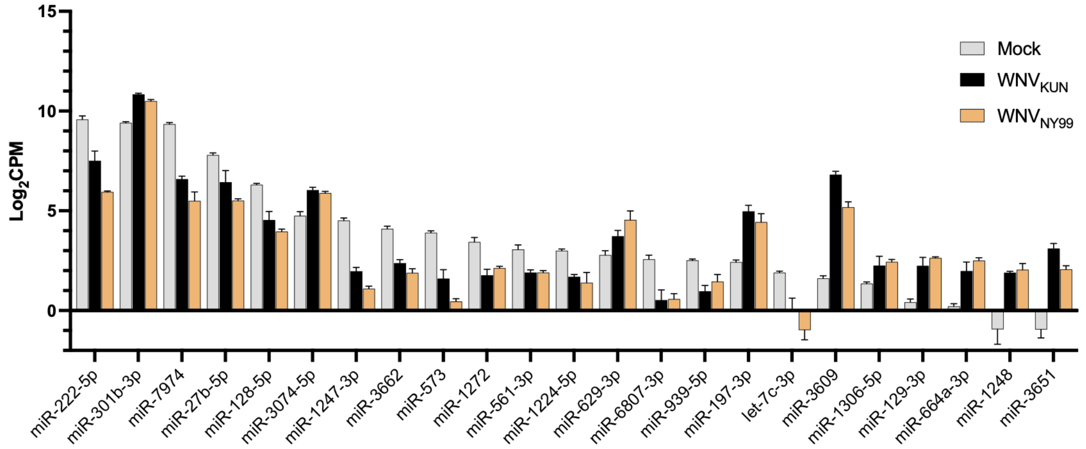

Supplementary figure S2. Expression levels of miRNA commonly differentially expressed in HEK-293 cells infected with WNV_{KUN} and WNV_{NY99}. The values are the means from th ree biological replicates ±SD; CPM – counts per million.

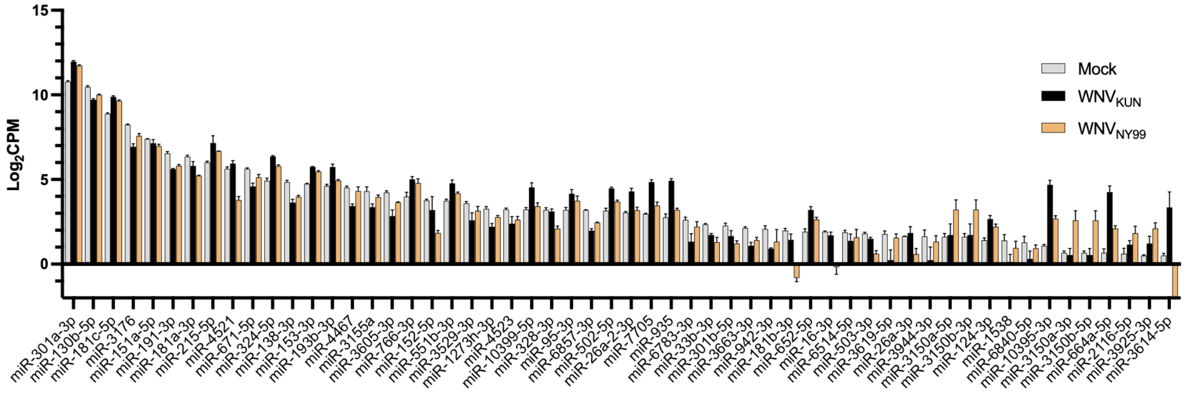

Supplementary figure S3. Expression levels of miRNA uniquely differentially expressed in HEK - 293 cells infected with either WNV_{KUN} or WNV_{NY99} compared to mock-infected cells. The val ues are the means from three biological replicates ±SD; CPM – counts per million.
